# Association Between Oral Health-Related Quality of Life and Depressive Symptoms in Older Adults in Canton Zurich: A Cross-Sectional Pilot Study

**DOI:** 10.3390/healthcare14162624

**Published:** 2026-08-19

**Authors:** Laura Rüegg, Sofya Sadilina, Eugenia Settecase, Ronald Ernst Jung, Murali Srinivasan, Tim Joda

**Affiliations:** 1 Clinic of Reconstructive Dentistry, Center for Dental Medicine, University of Zurich, Plattenstrasse 11, 8032 Zurich, Switzerland; laura.rueegg@uzh.ch (L.R.); sofya.sadilina@zzm.uzh.ch (S.S.); eugenia.settecase@zzm.uzh.ch (E.S.); ronald.jung@zzm.uzh.ch (R.E.J.); 2 Clinic of General, Special Care and Geriatric Dentistry, Center for Dental Medicine, University of Zurich, 8032 Zurich, Switzerland; murali.srinivasan@zzm.uzh.ch; 3 Center of Excellence in Precision Medicine and Digital Health, International Program in Geriatric Dentistry and Special Patients Care, Faculty of Dentistry, Chulalongkorn University, Bangkok 10330, Thailand; 4 Department of Reconstructive Dentistry, University Center for Dental Medicine Basel, University of Basel, 4058 Basel, Switzerland

**Keywords:** geriatric dentistry, gerodontology, older adults, oral health-related quality of life (OHRQoL), depression

## Abstract

**Objectives**: The objectives of this study were to assess the association between oral health-related quality of life (OHRQoL) and depressive symptoms among older adults in Canton Zurich, Switzerland, and to explore domain-specific variations. **Methods**: This pilot cross-sectional study included aging adults aged ≥65 years recruited from a university-based geriatric dental clinic in Zurich between 1 January and 31 March 2025. Participants completed paper-based questionnaires to assess their systemic and oral health, their quality of life (OHIP-14), and depressive symptoms (PHQ-8). Associations were assessed using Spearman’s rank correlation (ρ) with 95% confidence intervals estimated via bootstrap resampling. Domain-level analyses were conducted to explore the structure of the association, and a multivariable linear regression was used to assess whether the association persisted after adjustment. **Results**: A total of 108 participants were included (mean age of 76.2 ± 7.2 years; 53.4% male). Overall levels of OHRQoL and depressive symptoms were low. Higher OHIP-14 scores were moderately associated with higher PHQ-8 scores (ρ = 0.50; *p*-value < 0.01; 95% CI: 0.34–0.65). This association was consistent across PHQ-8 severity categories and remained significant after adjustment (β = 0.14; 95% CI: 0.06–0.22; *p* < 0.001). Domain-level analyses indicated stronger association in psychosocial and pain-related domains than for functional limitation. **Conclusions**: The study demonstrates that poorer OHRQoL was moderately associated with higher depressive symptoms, independently of the covariates examined, particularly in psychosocial domains. These findings suggest that OHRQoL reflects broader experiential and psychological dimensions of health beyond oral function alone, highlighting its relevance for oral health assessment in older adults. As the findings are based on a convenience sample of clinic attendees, larger population-based longitudinal studies are needed to clarify these relationships further.

## 1. Introduction

In 2021, approximately 761 million people worldwide were aged 65 or older, representing about 10% of the global population. By 2050, this number is expected to rise substantially and may reach 1.6 billion people [1]. In Switzerland, as in other developed countries, this demographic shift is accompanied by a growing emphasis of maintaining overall quality of life and well-being in older adults [2,3]. Among the factors influencing health aging, oral and mental health are increasingly recognized as important and potentially interrelated domains [4].

Mental disorders have become a leading cause of the global burden of disease, with depression among the most prevalent conditions, with lifetime estimates ranging from 6% to 21% [5,6,7,8]. According to the data from the Swiss Health Survey approximately 20% of individuals aged 65 and older experience depressive symptoms. These conditions are often underdiagnosed due to atypical clinical presentations and social stigma [9]. Risk factors such as social isolation, bereavement, chronic illness, and cognitive decline influence the onset, progression, and recurrence of depressive symptoms. Notably, many of these factors also influence the development and severity of oral diseases.

Oral diseases represent another major public health issue, affecting more than 3.5 billion people and accounting for the third highest treatment costs worldwide [10,11]. In older adults, oral health is a multifactorial issue influenced by lifetime oral hygiene habits, access to dental care, polypharmacy, and physical or cognitive impairments [2,12].

An increasing body of evidence supports a biopsychosocial model in which oral and mental health are closely interconnected [13]. Depression may lead to neglect of personal hygiene and reduced motivation for routine dental care [14,15]. Conversely, poor oral health may contribute to social withdrawal, pain, embarrassment, and nutritional issues, which in turn affect mood and increase the risk of depression [16].

To examine these interactions, the use of validated assessment tools is essential [17]. The Oral Health Impact Profile-14 (OHIP-14) provides a comprehensive measure of the impact of oral health on quality of life [18,19]. Similarly, the Patient Health Questionnaire-8 (PHQ-8) is a validated tool for screening depressive symptoms in the general population, including older adults. Understanding the relationship between these two assessments in older populations, particularly within the healthcare and socio-cultural context of Switzerland, may improve understanding of health vulnerabilities in aging communities and inform integrated preventive and public health strategies [20]. However, evidence on domain-specific association between oral health-related quality of life (OHRQoL) and depressive symptoms in older adults remains limited, particularly in Switzerland.

Given the influence of socio-economic and demographic factors, healthcare accessibility, and policy implications on oral health [6], the aim of this pilot cross-sectional study was to assess the association between OHRQoL and depressive symptoms in older adults in the Canton of Zurich, Switzerland, using the OHIP-14 and PHQ-8 scores, and to explore domain-specific variations. The study aimed to investigate the association between oral health-related quality of life, assessed using OHIP-14, and depressive symptoms, assessed using PHQ-8, among older adults in the canton of Zurich.

## 2. Methods

### 2.1. Study Design

This cross-sectional, single-center study was conducted at the Center for Dental Medicine, University of Zurich, Switzerland. This study was designed as an exploratory pilot investigation to estimate effect sizes and assess feasibility All assessments were performed by calibrated personnel working in the Clinic of General, Special Care and Geriatric Dentistry in the Center for Dental Medicine at the University of Zurich. The study adhered to the Strengthening the Reporting of Observational Studies in Epidemiology (STROBE) guidelines for cross-sectional studies [21,22]. The Cantonal Ethics Committee of Zurich confirmed that this study did not require formal ethical approval (BASEC-Nr. 2023-01621). All participants were informed about the study before providing the questionnaires, in accordance with the Declaration of Helsinki.

### 2.2. Participant Recruitment and Eligibility

Adults aged 65 years or more living in the Canton of Zurich and attending the Clinic of General, Special Care and Geriatric Dentistry in the Center for Dental Medicine at the University of Zurich, Switzerland, were recruited between 1 January and 31 March 2025. Participants were recruited using a convenience sampling approach. The exclusion criteria included being under 65 years old and insufficient German language proficiency. Potential participants received written information about the study. They were informed that participation was voluntary and that they could decline to participate without any consequences for their treatment at the clinic. Participants were also informed that they could contact the project leader at any time if they had questions regarding the study.

### 2.3. Data Collection

Data was collected using three standardized, paper-based questionnaires. All questionnaires were distributed to patients in paper form and were completed independently or with the assistance of trained study personnel, if required. Assistance was limited to clarification of questions without influencing responses [23,24]. Study personnel helped when needed and collected the questionnaire immediately after completion. The questionnaire took between 10 and 15 min. The investigated survey consists of the following components:

- General systemic health questionnaire

This questionnaire assessed participants’ systemic health and level of self-reliance. The full instrument is provided in Figure S1.

- Oral health-related quality of life (OHRQoL)

OHRQoL was evaluated using the validated German 14-item version of the Oral Health Impact Profile (OHIP-14G) [18,25]. Items cover functional limitations, physical pain, psychological discomfort, psychological disability, social disability, and handicap. Responses were recorded on a five-point Likert scale (0 = “never” to 4 = “very often”). Total OHIP-14 scores range from 0 to 56, with higher scores indicating poorer quality of life. Participants also self-reported the number of remaining teeth. The instrument is reproduced in Figure S2.

- Depressive symptoms

Depressive symptoms were evaluated using the 8-item version of the Patient Health Questionnaire (PHQ-8) [26]. PHQ-8 measures the frequency of depressive symptoms over the past two weeks across 8 items. Responses are recorded on a four-point scale ranging from ‘not at all’ (0 points) to ‘nearly every day’ (3 points). The total scores ranged between 0 and 24, with a higher score indicating greater severity of depression symptoms. A score of ≥10 was indicative of clinically elevated levels of depressive symptoms [27]. The questionnaire is listed in Figure S3. Both instruments have been validated in German-speaking populations. All responses were anonymized at the time of collection. No personal identifiers were recorded to preserve patient confidentiality, maintain blinding, and minimize potential sources of bias.

### 2.4. Sample Size

The participants were older adults who were patients of a university-based geriatric dental clinic in Zurich, Switzerland. Given the pilot nature of this study, no formal priori sample size calculation was performed. The primary objective was to estimate effect sizes and assess feasibility of future research. The participants were recruited using a convenience sampling approach from clinic-attending older adults; therefore, the sample was not population-based. The final sample size (*n* = 108) was considered sufficient for an exploratory correlation analysis and estimation of confidence intervals. In addition, the study aimed to generate preliminary estimates to inform sample size calculations for confirmatory epidemiological research.

Due to the limited sample size available for multivariable analysis, the adjustment set was specified a priori and restricted to covariates with the clearest conceptual relevance to the OHIP-14–PHQ-8 association and, where appropriate, evidence of association in univariable analyses. This parsimonious specification was intended to limit model complexity and reduce the risk of overfitting and unstable coefficient estimates relative to the available sample size.

### 2.5. Statistical Analysis

Paper-based forms were collected and transcribed into a structured Microsoft Excel database (Microsoft Corporation, Redmond, WA, USA, 2024) using predefined drop-down menus to minimize data entry errors. All statistical analyses were performed using R Statistical Software (version 4.4.1; R Core Team).

Descriptive statistics were used to summarize participant characteristics. Continuous variables were reported as mean and standard deviation (SD) or median and interquartile range (IQR), depending on data distribution. Categorical variables were summarized as frequencies and percentages.

The primary analysis explored the association between oral health-related quality of life (OHIP-14) and depressive symptoms (PHQ-8) by calculating the non-parametric Spearman’s correlation coefficient (ρ), given that the variables to evaluate were ordinal. Ninety-five-percent confidence intervals (95% CIs) were estimated through bootstrap resampling (2000 iterations) [28]. To assess whether the association between OHIP-14 and PHQ-8 persisted after adjusting for potential confounders, we conducted a secondary, confirmatory multivariable linear regression analysis.

Domain-level analyses were conducted by grouping OHIP-14 items into conceptual domains and correlations with individual PHQ-8 items were visualized using heatmaps. Furthermore, OHIP-14 scores were compared across PHQ-8 severity categories using boxplots. Given the exploratory nature of the study, the analysis focused on effect size, magnitude, and direction rather than statistical significance. Missing data were handled using complete-case analysis, as the proportion of missing data was low and no systematic pattern were identified.

## 3. Results

### 3.1. Participants’ Characteristics

A total of 108 participants, who met the inclusion criteria, were included in the study. Of these, 53.4% were male and 46.6% were female. The participants’ ages ranged from 65 to 95 years, with a mean age of 76.2 years (SD 7.2). A high majority of the patients (90.7%) reported that they visited a physician within the previous year. In terms of oral status, 6.8% of responders were fully edentulous, while the majority, 83.5%, had a limited number of natural teeth, ranging from one to six or more, but not all. Nearly half of the clinic-attending participants reported that they did not use removable dentures (47.1%), while partial removable dentures were the most common prosthesis used (45.1%). The full demographic and systemic health characteristics of the sample are presented in Table 1.

Across the study variables, the proportion of missing observations ranged from 0.0% to 15.7%, with 9.1% of all variable-level observations missing overall. Missing data was primarily due to incomplete questionnaire responses, which are common in survey-based [29]. No systematic pattern was found in the missing values. The complete case analysis resulted in the exclusion of 34 participants from the relevant analyses. Given the exploratory nature of the study and the relatively low proportion of missing data, no imputation was performed.

### 3.2. Questionnaire Outcomes

For self-reported oral health status using the OHIP-14 scale, the median OHIP-14 score was 6 points (IQR 15). Over half of the clinic-attending participants (52.0%) had minimal scores (0–6), while 33.3% had a mild score (7–21), and 14.7% showed a severe OHIP score (>21).

The self-reported mental depression score, measured using the PHQ-8, had a median PHQ-8 score of 3 points (IQR 5). Most participants (67.0%) showed no or minimal depression (0–4), followed by those presenting mild depression (5–9) (25.0%). The rest of the participants presented either moderate depression (9–14) (4.0%), moderately severe depression (15–19) (3.0%), or severe depression (>19) (1.0%). The distribution of responses to individual questionnaire items is presented in Supplementary Materials (Figures S4 and S5).

**Figure 1:**
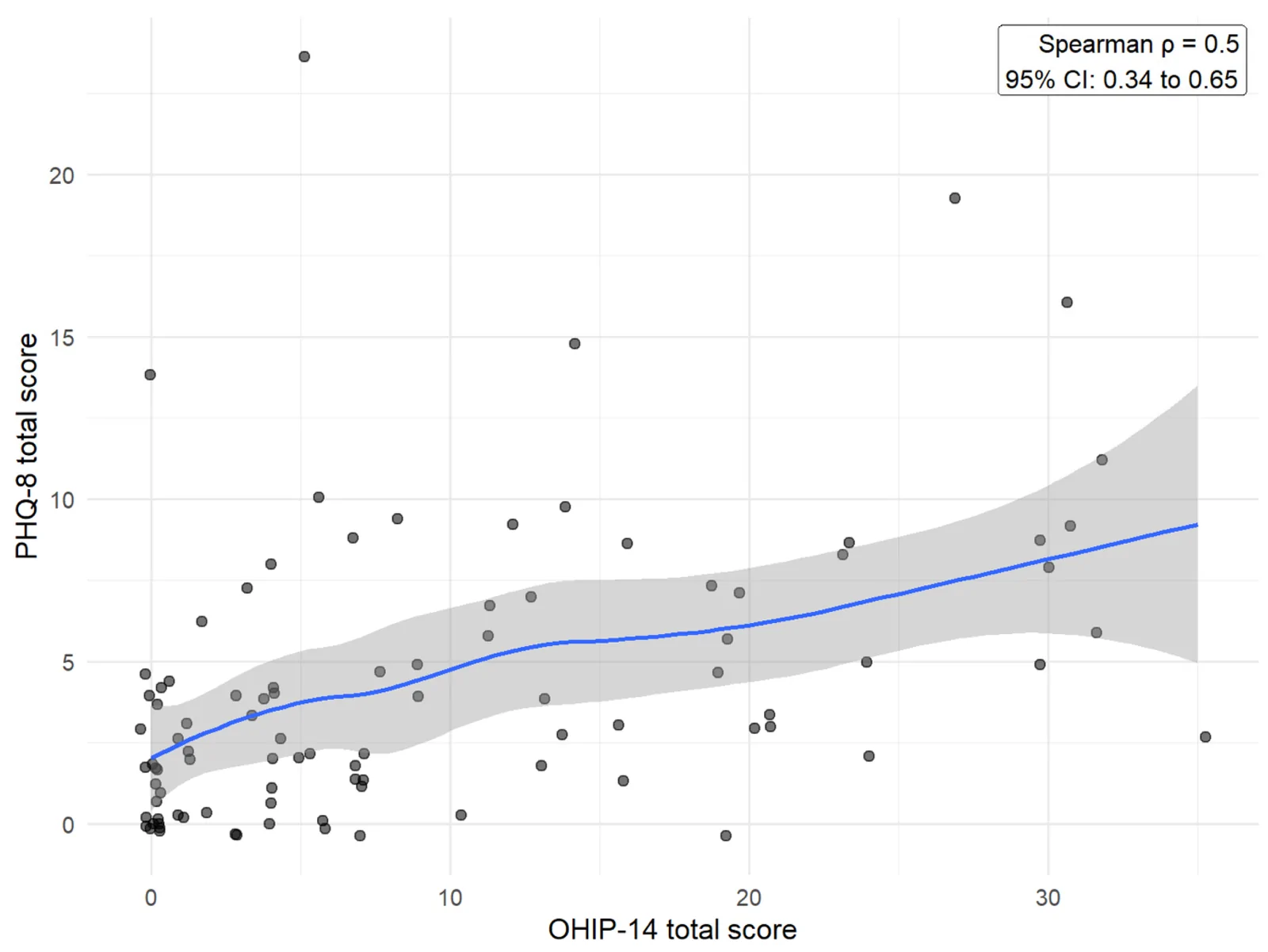
Association between oral health-related quality of life (OHIP-14) and depressive symptoms (PHQ-8) (*n* = 95). Each point represents one participant. The blue line shows a LOESS-smoothed trend, with the shaded band indicating its 95% confidence interval; the boxed statistic reports the overall Spearman correlation coefficient and its separate 95% bootstrap confidence interval.

A moderate positive correlation was observed between PHQ-8 and OHIP-14 scores (Spearman’s ρ = 0.50; *p*-value < 0.01; 95% CI: 0.34–0.65), indicating that poorer oral health-related quality of life was correlated with higher levels of depressive symptoms (Figure 1). Bootstrap confidence intervals (95% CI: 0.34; 0.65) indicated that this association was consistently positive and of moderate magnitude, supporting the robustness of this relationship.

#### 3.2.1. Multivariable Analysis

To assess whether the association between OHIP-14 and PHQ-8 persisted after adjustment for potential confounders, a multivariable linear regression was conducted using a pre-specified covariate set (age, gender, and self-reported psychological distress; Table S1). This parsimonious specification was intended to limit model complexity and reduce the risk of overfitting and unstable coefficient estimates relative to the available sample size. After adjustment, the association between OHIP-14 and PHQ-8 remained significant (β = 0.14 < 95% CI: 0.06–0.22; *p* < 0.001), indicating that a one-point increase in OHIP-14 score was associated with a 0.14-point increase in PHQ-8 score, independently of the covariates examined. Self-reported psychological distress was independently associated with higher PHQ-8 scores (β = 4.25; *p* = 0.001), while male gender was associated with lower PHQ-8 scores (β = −1.52; *p* = 0.050). The covariate age was not significant. For sensitivity analyses, an additional full model adjusting for all measured demographic and clinical covariates (age, gender, diabetes, self-reported psychological distress, smoking status, alcohol consumption, number of own teeth, and denture status) was examined. This produced estimates consistent with the prior model (Table S2). Together, these sensitivity analyses support the robustness of the OHIP-14–PHQ-8 association across a range of covariate specifications.

All regression coefficients were computed using the boot package in R, with 95% bootstrap confidence intervals (2000 resamples) being used to account for mild non-normality in model residuals (Tables S1 and S2).

#### 3.2.2. Severity-Category Breakdown

**Figure 2:**
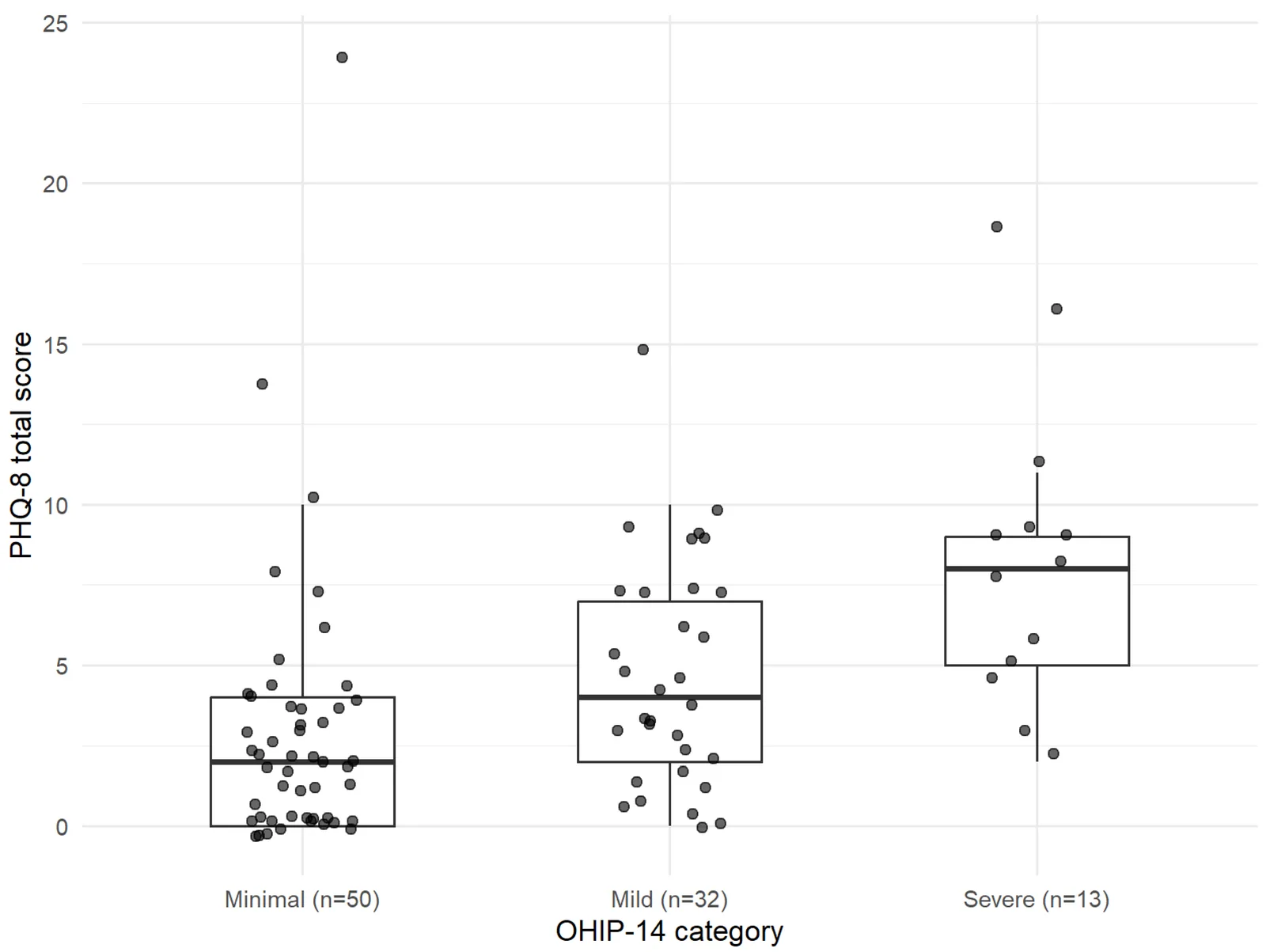
Distribution of Patient Health Questionnaire-8 (PHQ-8) score across Oral Health Impact Profile-14 (OHIP-14) score (minimal, mild, severe).

When OHIP-14 scores were examined across PHQ-8 severity categories, a clear pattern emerged: participants with higher levels of depressive symptoms tended to report worse oral health-related quality of life (Figure 2). This trend was observed across all levels of symptom severity.

**Figure 3:**
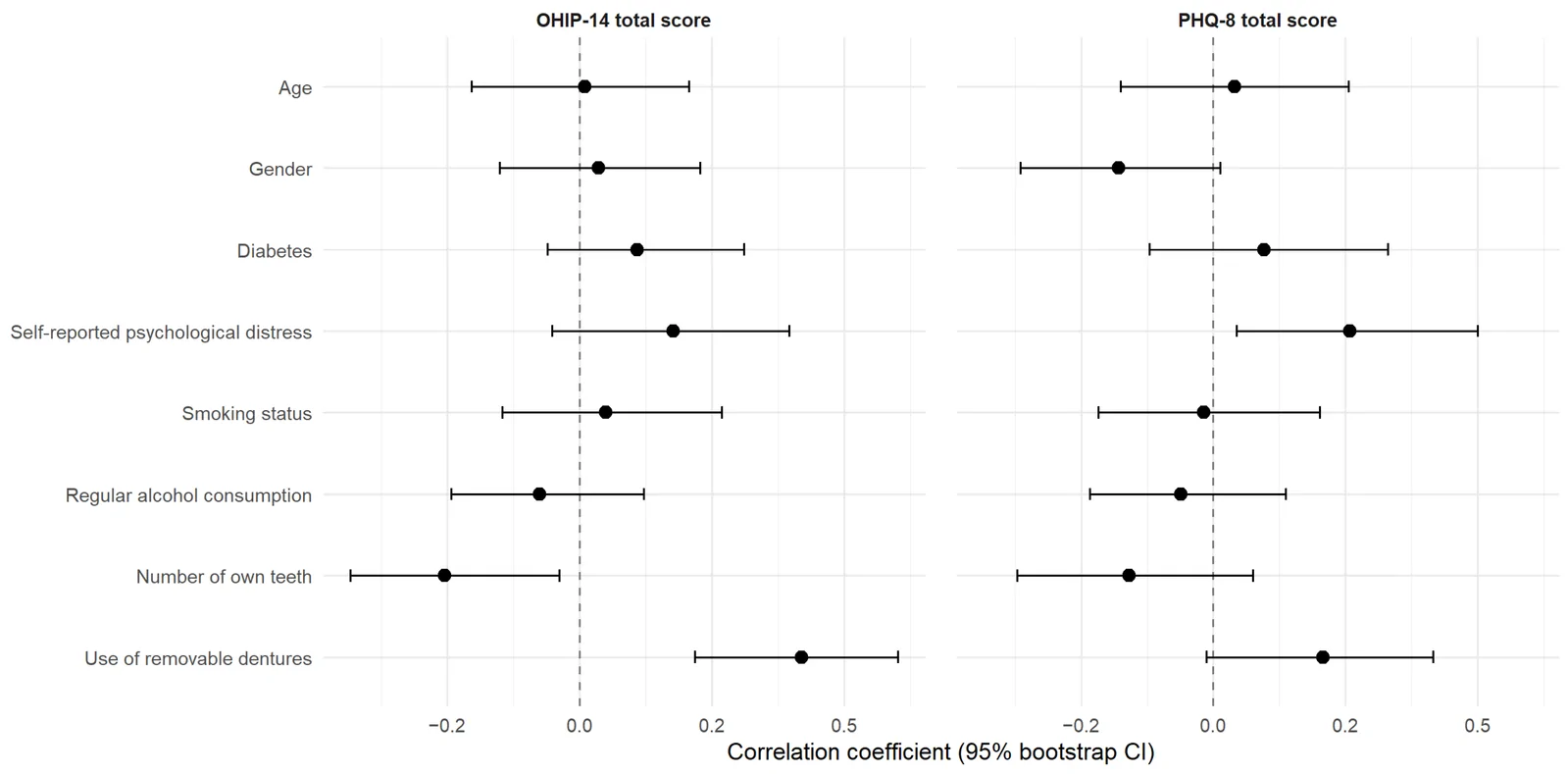
Associations between demographic/clinical variables and OHIP-14 and PHQ-8 total scores. Point-biserial correlations (r) are shown for binary variables (gender, diabetes, self-reported psychological distress, smoking status, alcohol consumption); Spearman correlations (ρ) are shown for age and ordinal oral-status variables (number of own teeth, denture status).

Exploratory analyses showed that the association between OHIP-14 and PHQ-8 was among the strongest observed in the dataset. A poorer dental status was related to greater oral health-related impacts. Participants with partial or complete dentures had higher OHIP-14 scores than those without dentures, while a greater number of natural teeth own was associated with lower OHIP-14 scores. In contrast, relationships with demographic and clinical variables (such as age, smoking, alcohol use, diabetes, and denture use) were generally weak and imprecise, with effect sizes close to zero (Figure 3).

**Figure 4:**
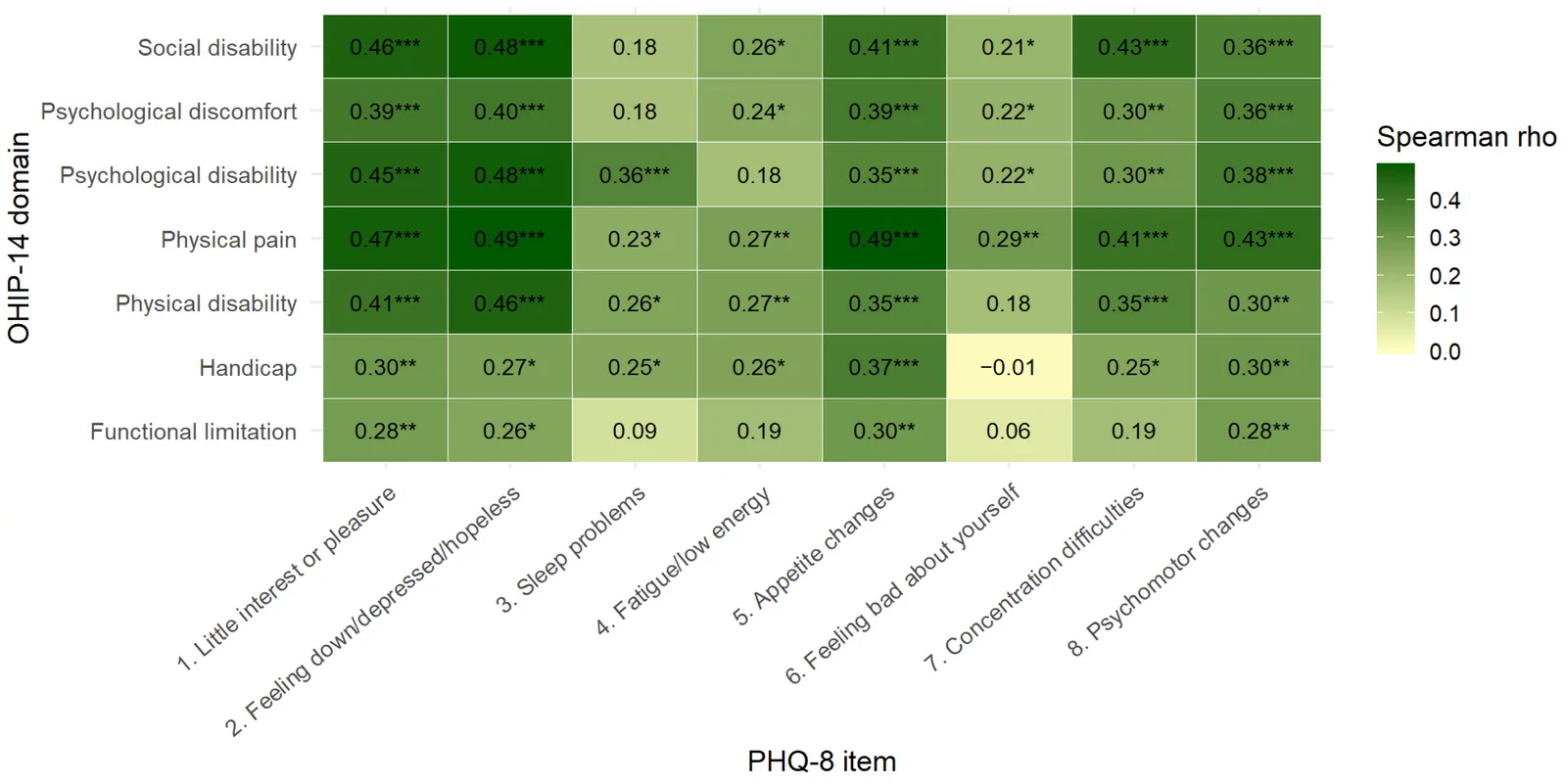
Spearman correlations between OHIP-14 domain scores and individual PHQ-8 items (*n* = 108). PHQ-8 item wording is shown on the *x*-axis. Asterisks denote statistical significance (* *p* < 0.05, ** *p* < 0.01, *** *p* < 0.001); unmarked cells are not statistically significant.

Domain-level analyses further demonstrated that the association between OHIP-14 and depressive symptoms was not consistent across the different components of OHRQoL (Figure 4). Stronger correlations were observed in domains related to physical pain, psychological discomfort, psychological disability, and social disability, while functional limitation showed comparatively weaker associations with PHQ-8 items. This pattern suggests that the overall association between OHIP-14 and PHQ-8 is driven primarily by psychosocial and experiential factors rather than by functional impairment alone.

At the item level, correlations between individual PHQ-8 questions and OHIP domains were generally consistent in direction but varied in magnitude. Moderate correlations were particularly observed for items related to mood, fatigue, and loss of interest (Figure 4). These findings suggest that the overlap between OHRQoL and depressive symptoms is distributed across multiple symptom domains, rather than being driven by a single component.

Overall, these analyses demonstrate that, although a moderate association exists between OHIP-14 and PHQ-8, this relationship is structured and domain-specific. It is also more strongly aligned with the psychosocial dimensions of OHRQoL than with functional or systemic health indicators.

## 4. Discussion

The present pilot cross-sectional study explored the association between OHRQoL and depressive symptoms among older adults attending a university-based geriatric dental clinic in Zurich, Switzerland. A moderate positive association was observed between OHRQoL measured by OHIP-14 and depressive symptoms measured by PHQ-8 (ρ = 0.50; *p*-value < 0.01; 95% CI: 0.34–0.65), indicating that poorer OHRQoL was associated with higher levels of depressive symptoms. The consistency of this association across the range of values, as well as across PHQ-8 severity categories, supports the robustness of the observed relationship. Importantly, domain-level analyses demonstrated that this association was not uniform across dimensions of OHRQoL but was stronger in psychosocial disability and pain-related domains than in functional limitation.

A reduced multivariable linear regression was conducted over a pre/selected number of covariates due to the small sample size of the study. The results showed that a one-point increase in OHIP-14 score was associated with a 0.14-point increase in PHQ-8 score (95% CI: 0.06–0.22; *p* < 0.001), indicating that the relationship between poorer OHRQoL and greater depressive symptoms persists independently of the examined covariates. Given the mild deviations from normality observed in the model residuals, bootstrap confidence intervals were obtained and the results were consistent, which supports the robustness of this estimate. Self-reported mental illness was independently associated with higher depressive symptom scores. A full model was adjusted as a sensitivity analysis, in which it was observed that oral health-related clinical variables, such as denture status and the number of teeth lost, showed no independent association with the PHQ-8 once OHRQoL was accounted for. This suggests that self-reported OHRQoL may capture aspects of the psychological burden that are not reflected in more objective oral health indicators.

It is also important to consider the potential overlap between the psychosocial domains of OHIP-14 and the PHQ-8 symptom items. Domains such as psychological discomfort and psychological disability cover concepts such as self-consciousness, embarrassment and reduced life satisfaction, which are like core depressive symptoms such as low mood and negative self-evaluation. As these domains were identified as the main drivers of the OHIP-14–PHQ-8 association, some of the correlation may be due to the measurement of general psychological distress rather than an oral health-mediated pathway to depressive symptoms. Nevertheless, the persistence of the association after adjusting for demographic and clinical covariates, together with the comparatively weaker associations observed for the functional and physical disability domains, suggests that redundancy alone is not the sole factor in the relationship.

Population aging represents a well-documented trend in developed countries. As life expectancy increases, maintaining both oral health and mental well-being becomes increasingly important for preserving quality of life in older age [1,20]. In Switzerland, a well-developed healthcare system provides an opportunity to study these dynamics in a relatively well-supported population, although inequality still exists, particularly among rural older adults and those in long-term care facilities [2,30,31]. Therefore, the present findings should be interpreted within the context of this relatively healthy and more resource-supported cohort [32].

The findings of the study are consistent with previously published studies reporting associations between poor oral health and depressive symptoms across different countries, including Germany, Japan, and the United States [33,34]. A meta-analysis has also confirmed this correlation in older adults, although effect sizes very and causal pathways remains unclear [35]. The current study extends this evidence by suggesting that the association is structured and domain-specific rather than uniform across all aspects of OHRQoL.

From a community dentistry perspective, these findings suggest that OHRQoL reflects not only clinical oral status but also broader psychosocial and social determinants of health. The observed association between OHRQoL and depressive symptoms highlights the potential influence by factors such as social isolation, psychological distress, and access to care. This supports the need of population-based approaches that consider oral health within the wider context of general health and social conditions.

From a demographic perspective, the study aligns with trends reported in the literature for older adults. Participants represented an aged sample with a relatively high number of natural teeth (68.0%), i.e., six or more, but not all. This reflects improvements in tooth retention in recent decades. Nevertheless, more than half of the participants (52.9%) wore partial or complete removable dentures, indicating that tooth loss remains a significant concern at an advanced age [36,37]. Despite Switzerland’s well-developed healthcare infrastructure, these findings highlight persistent oral health challenges in older adults [38].

This study has several limitations that should be considered when interpreting the findings. First, the cross-sectional design prevents any inference about causality or directionality. Second, using a convenience sample size of clinic-attending older adults may introduce selection bias and limits the generalizability to the broader population. For example, these individuals may be more independent, have better access to public transport and be more proactive in seeking healthcare. Those seeking dental care may differ from the general older adult population in terms of their oral health status, and individuals experiencing the most severe depressive symptoms or a greater degree of social isolation may be underrepresented. The direction and magnitude of the resulting bias on the observed OHIP-14–PHQ-8 association cannot be determined from the present cross-sectional data, but the findings should be interpreted with this selection process in mind. Third, the relatively small sample size and single-center design may limit external validity, and the multivariable analysis was based on a reduced subsample (*n* = 74 participants) due to complete-case requirements across covariates, with limited representation of some subgroups such as self-reported mental illness (*n* = 11). Fourth, reliance on self-reported measures may introduce reporting bias and shared method variance. Fifth, as discussed above, potential conceptual overlap between OHIP-14 psychosocial domains and PHQ-8 items may have contributed to the strength of the observed association.

Despite these limitations, a key strength of this study is the use of validated and widely applied instruments (OHIP-14 and PHQ-8), standardized data collection procedures, and detailed domain-level analysis that provide a more nuanced understanding of association between oral health-related quality of life and depressive symptoms.

Given the aging population, the relationship between oral health and depression has important implications for public health policy and healthcare resource allocation. Comprehensive measurements targeting both oral and mental health could improve quality of life and reduce healthcare costs in older adults. However, as this study was conducted within a single healthcare and insurance system in Switzerland, caution is required when extrapolating findings to other healthcare systems or more socioeconomically diverse populations.

Future research should include larger, population-based and longitudinal studies to clarify potentially bidirectional relationship between OHRQoL and depressive symptoms and to explore these associations in different healthcare and insurance systems. Studies employing instruments with stronger discriminant validity from depression measures, or latent-variable approaches such as confirmatory factor analysis, would help determine the extent to which this association reflects a genuine cross-domain relationship rather than overlapping item content. Special attention should be given to high-risk groups, such as those with cognitive impairment or limited access to care, where both oral health and mental burdens are typically higher.

## 5. Conclusions

This pilot cross-sectional study demonstrated a moderate association between oral health-related quality of life and depressive symptoms among older adults in Canton Zurich. The association was not uniform across all of OHRQoL domains, being stronger in the psychosocial and pain-related domains than in the functional components. It persisted even after adjusting for demographic, health characteristics, and participant habits covariates. These findings suggest that OHRQoL reflects broader psychological dimensions of health beyond oral function alone. The domain-specific pattern observed also raises the possibility of constructing overlaps with measures of depressive symptoms. In many countries, including Switzerland, dental medicine remains separate from systemic healthcare, with dental care falling outside mandatory basic health insurance. The present findings suggest that closer integration of oral and mental healthcare, for example through preventive dental strategies, may be valuable not only for OHRQoL, but also for downstream mental health outcomes and associated healthcare costs. However, given the observational design of this study, this possibility should be regarded as a hypothesis for future health economic and longitudinal research. Taken together, the results highlight the importance of integrating oral and mental health assessments in aging populations. However, given the cross-sectional exploratory design, the current findings should be interpreted cautiously and may not generalize to older adults who do not access dental care or general healthcare. Further research is needed in the form of larger, population-based and longitudinal studies to clarify these relationships in more representative samples.

## Figures and Tables

**Table 1:** Characteristics of study sample (*n* = 108).

Variable	Value	Number of Available Answers
Demographics		
Age in years, mean ± SD	76.2 ± 7.2	103
Gender, *n* (%)		103
Female	48 (46.6)	
Male	55 (53.4)	
Medical card/passport holder, *n* (%)		106
No	78 (73.6)	
Yes	28 (26.4)	
Last physician visit, *n* (%)		107
Within the past year	97 (90.7)	
One year ago or more	10 (9.3)	
Health characteristics		
Diabetes, *n* (%)		102
No	79 (77.5)	
Yes	23 (22.5)	
Self-reported psychological distress, *n* (%)		101
No	90 (89.1)	
Yes	11 (10.9)	
Smoking status, *n* (%)		107
Non-smoker	83 (77.6)	
Current smoker	24 (22.4)	
Regular alcohol consumption, *n* (%)		107
No	79 (73.8)	
Yes	28 (26.2)	
Oral characteristics		
Number of own teeth ^†^, *n* (%)		103
0	7 (6.8)	
3	16 (15.5)	
16.5	70 (68.0)	
28	10 (9.7)	
Use of removable dentures, *n* (%)		102
Complete	8 (7.8)	
Partial	46 (45.1)	
None	48 (47.1)	
History of dental trauma, *n* (%)		107
No	95 (88.8)	
Yes	12 (11.2)	
Questionnaire outcomes		
OHIP-14 score, mean ± SD *	9.6 ± 10.2	102
OHIP-14, *n* (%)		102
Minimal	53 (52.0)	
Mild	34 (33.3)	
Severe	15 (14.7)	
PHQ-8 score, mean ± SD **	4.1 ± 4.4	100
PHQ-8, *n* (%)		100
No or minimal depression	67 (67.0)	
Mild depression	25 (25.0)	
Moderate depression	4 (4.0)	
Moderately severe depression	3 (3.0)	
Severe depression	1 (1.0)	

^†^ The number of own teeth was approximated numerically from these categories using midpoint values: none = 0, 1–5 = 3, 6 or more (but not all) = 16.5; all = 28. Abbreviations: OHIP-14, Oral Health Impact Profile-14, PHQ-8, Patient Health Questionnaire-8, SD, standard deviation. * The Oral Health Impact Profile-14 (OHIP-14) summary score ranges from 0 to 56, with a lower score representing better oral health-related quality of life. ** The Patient Health Questionnaire-8 (PHQ-8) summary score ranges from 0 to 24, with a lower score indicating fewer depressive symptoms.

## Data Availability

The datasets used in the study are available from the corresponding author upon reasonable request. The data are not publicly available due to considerations regarding privacy, ethical oversight, and the controlled sharing of clinical data.

## Supplementary Materials

The following supporting information can be downloaded at https://www.mdpi.com/article/10.3390/healthcare14162624/s1. Figure S1: Example of questionnaire assessing participants’ systemic health and level of self-reliance. Figure S2: Example of German 14-item version of the Oral Health Impact Profile (OHIP-14G). Figure S3: Example of Patient Health Questionnaire-8 (PHQ-8), version in German. Figure S4: Distribution of responses to the Oral Health Impact Profile-14 (OHIP-14) items among the study participants. Figure S5: Distribution of responses to the Patient Health Questionnaire-8 (PHQ-8) items among the study participants. Table S1: Multivariable linear regression of PHQ-8 score on OHIP-14 score, adjusted for gender and self-reported psychological distress (*n* = 74). Table S2: Multivariable linear regression of PHQ-8 score on OHIP-14 score adjusted for the full covariate set, presented as an exploratory sensitivity analysis.
