# Supplementary material for: Association Between Oral Health-Related Quality of Life and Depressive Symptoms in Older Adults in Canton Zurich: A Cross-Sectional Pilot Study
Source: Healthcare (Basel). 2026 Aug 19;14(16):2624. doi: 10.3390/healthcare14162624 (PMC13512465; doi:10.3390/healthcare14162624)
Supplement: Supplementary file 1 [file healthcare-14-02624-s001.zip › healthcare-4470002-supplementary/healthcare-4470002-supplementary.pdf]

Supplementary materials

University of Zurich[UZH]

Zentrum für Zahnmedizin
Klinik für Rekonstruktive Zahnmedizin

Direktor: Prof. Dr. med. dent. Ronald Jung, PhD
Plattenstrasse 11
CH-8032 Zürich
Telefon +41 44 634 34 53
Telefax +41 44 634 43 05
www.zzm.uzh.ch

Subject No. ☐☐☐☐ Date ☐☐.☐☐.☐☐☐☐

Information on General Health Status

Yes	No		
		1	Have you been vaccinated against tetanus? When _____________
☐	☐	2	Have you ever had an unusual reaction (allergy, etc.) to injections, medications, or dental materials?
☐	☐	3	Do you have a medical ID card/pass<br>(antibiotic precautions, blood thinners, transplants, joint replacements, pacemaker)?
☐	☐	4	Have you been examined by a doctor within the past year?
☐	☐	5	Have you ever been seriously ill and/or hospitalized?
☐	☐	6	Do you bleed easily or for a long time when you get injured?
☐	☐	7	Have you ever had an accident involving your face or jaw, or have you had surgery or radiation therapy in that area?
☐	☐	8	Have you ever had an accident that resulted in injury to your teeth?
☐	☐	9	Do you currently suffer from, or have you ever suffered from, any of the following conditions:
☐	☐	a)	Asthma or hay fever?
☐	☐	b)	Shortness of breath during mild exertion (climbing stairs)?
☐	☐	c)	Diabetes?
☐	☐	d)	Heart disease?
☐	☐	e)	High/low blood pressure?
☐	☐	f)	Infections (tuberculosis, hepatitis, sexually transmitted diseases, HIV/AIDS)?
☐	☐	g)	Osteoporosis?
☐	☐	h)	Mental distress?
☐	☐	i)	Cancer?
☐	☐	j)	Other: ____
☐	☐	10	Do you faint easily?
☐	☐	11	Do you suffer from frequent stomach or digestive problems, or do you vomit often?
☐	☐	12	Do you smoke? If so, how much? ______________________________<br>For how many years? _____________________________________
☐	☐	13	Do you drink alcohol regularly?<br>If so, how much? ____
☐	☐	14	Do you regularly use soft or hard drugs? Which ones___________

Figure S1. Example of questionnaire assessed participants' systemic health and level of self-reliance.

Subject No. ☐☐☐☐ Date ☐☐.☐☐.☐☐☐☐

PROMs: Questionnaire on the Number of Natural Teeth

How many of **your own permanent teeth** do you have in your mouth?

☐ None

☐ 1 to 5

☐ 6 or more (but not all)

☐ All

Do you have **removable dentures** (false teeth) that you wear? (Crowns and bridges do not count.)

☐ No

☐ I have my own teeth and also wear a removable denture in one or both jaws.

☐ I am edentulous and have full dentures in both the upper and lower jaws.

PROMs: Oral Health Questionnaire (OHIP-14G)

**In the past 7 days, because of problems with your teeth, mouth, jaw, or dentures, have you...**					
	very often 4	often 3	occasionally 2	hardly ever 1	never 0
Difficulty pronouncing certain words?					
Did you feel like your sense of taste was impaired?					
the impression that your life was generally less satisfying?					
Difficulty relaxing?					

PROMs: Fragebogen zur Mundgesundheit (OHIP-14G) [*Fortsetzung*]

**In the past 7 days, because of problems with your teeth, mouth, jaw, or dentures, has it happened that...**					
	very often 4	often 3	occasionally 2	hardly ever 1	never 0
Did you feel tense?					
that you had to stop eating in the middle of a meal?					
that you felt uncomfortable eating certain foods?					
that you've been more irritable toward other people?					
that you found it difficult to carry out your daily activities?					
that you were completely unable to do anything?					
Did you feel a little embarrassed?					
that your diet has been unsatisfactory?					

**In the past 7 days, have you …**					
	very often 4	often 3	occasionally 2	hardly ever 1	never 0
Pain in your mouth?					
A feeling of uncertainty regarding your teeth, your mouth, or your dentures?					

Figure S2. Example of German 14-item version of the Oral Health Impact Profile (OHIP-14G).

University of Zurich[UZH]

Zentrum für Zahnmedizin
Klinik für Rekonstruktive Zahnmedizin

Direktor: Prof. Dr. med. dent. Ronald Jung, PhD
Plattenstrasse 11
CH-8032 Zürich
Telefon +41 44 634 34 53
Telefax +41 44 634 43 05
www.zzm.uzh.ch

Subject No. ☐☐☐☐ Date ☐☐.☐☐.☐☐☐☐

The 8-Item Patient Health Questionnaire for Depression (PHQ-8)

**How often have you been bothered by the following problems in the past 14 days?**				
	Never (0–1 days)	Sometimes (2–6 days)	Often (7–11 days)	Very often (12–14 days)
1. Little interest in or enjoyment of doing things				
2. Feeling down or hopeless				
3. Trouble falling asleep or staying asleep, or sleeping too much				
4. Fatigue or low energy				
5. Loss of appetite or overeating				
6. Feeling bad—or that you're a failure or have let yourself or your family down				
7. Difficulty concentrating, for example while reading the newspaper or watching TV				
8. Moving or speaking so slowly that others might not have noticed. Or the opposite—feeling fidgety or restless because you've been moving around much more than usual				

Kroenke K, Strine TW, Spitzer RL, Williams JB, Berry JT, Mokdad AH.
The PHQ-8 as a measure of current depression in the general population.
J Affect Disord. 2009;114(1-3):163-73.

Figure S3. Example of Patient Health Questionnaire – 8 (PHQ-8), version in German.

Table S1. Multivariable linear regression of PHQ-8 score on OHIP-14 score, adjusted for, gender and self-reported psychological distress (n = 74).

Variable	Estimate	SE	95% IC		p-value	Boot 95% IC	
			lower	upper		lower	upper
Intercept	2.76	0.61	1.54	3.97	< 0.001	-4.57	11.74
OHIP-14 score	0.14	0.04	0.06	0.22	< 0.001	0.07	0.22
Age	0.006	0.52	-0.10	0.11	0.91	-0.11	0.09
Gender	-1.45	0.72	-2.89	-0.01	0.050	-2.88	-0.10
Self-reported psychological distress	4.25	1.24	1.77	6.72	0.001	1.58	8.61

Table S2. Multivariable linear regression of PHQ-8 score on OHIP-14 score adjusted for the full covariate set, presented as an exploratory sensitivity analysis.

Variable	Estimate	SE	95% IC		p-value	Boot 95% IC	
			lower	upper		lower	upper
Intercept	0.62	4.57	-8.50	9.74	0.89	-6.67	11.01
OHIP-14 score	0.15	0.04	0.06	0.23	< 0.001	0.08	0.25
Age	0.03	0.06	-0.08	0.14	0.58	-0.10	0.12
Gender	-1.52	0.85	-3.23	0.19	0.08	-3.28	0.17
Diabetes	1.09	0.92	-0.75	2.93	0.24	-0.57	3.73
Self-reported psychological distress	3.87	1.33	1.21	6.53	< 0.01	1.42	9.07
Regular alcohol consumption	0.45	0.98	-1.51	2.41	0.65	-1.39	2.44
Smoking status	1.09	0.87	-2.44	1.06	0.43	-2.58	1.00
Number of lost teeth	0.00	0.06	-0.12	0.12	0.98	-0.13	0.10
Use of removable dentures – Partial	-0.91	0.92	-2.74	0.92	0.32	-3.34	0.71
Use of removable dentures - Complete	0.29	1.76	-3.24	3.81	0.87	-3.39	5.28

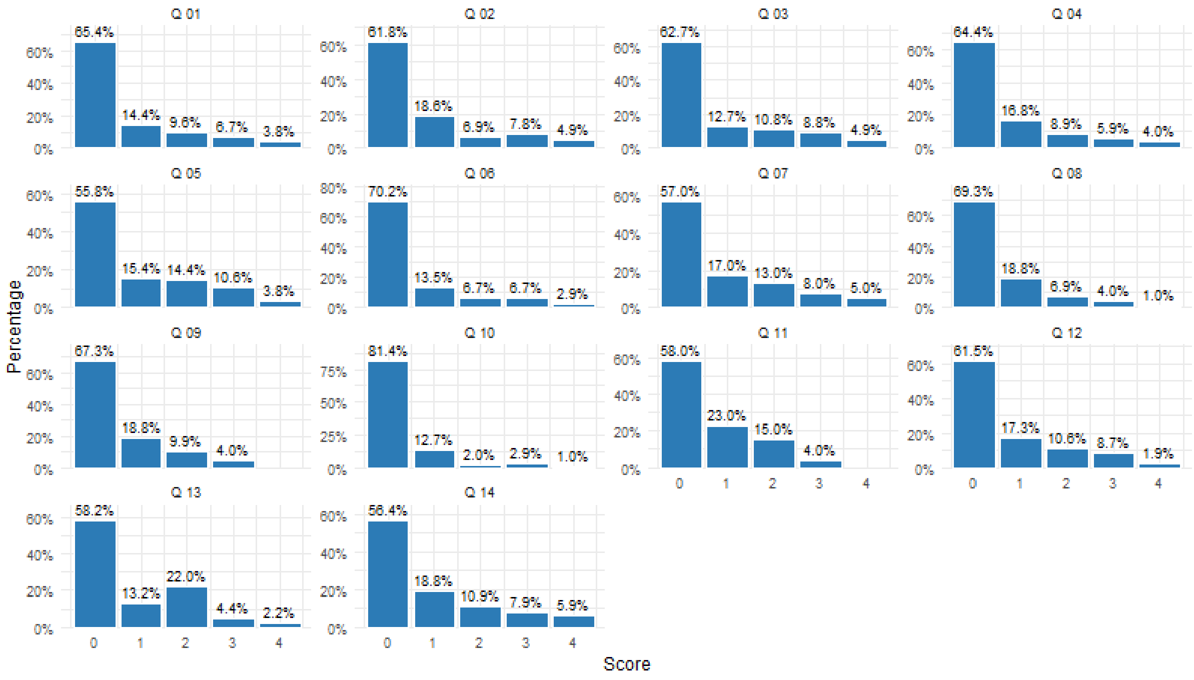

Abbreviations: Q, question.

Figure S4. Distribution of responses to the Oral Health Impact Profile-14 (OHIP-14) items among the study participants. Each panel represents one OHIP-14 item (Q1–Q14), and bars indicate the percentage of participants reporting each score (0 = never to 4 = very often). Higher scores reflect a greater negative impact of oral health on quality of life.

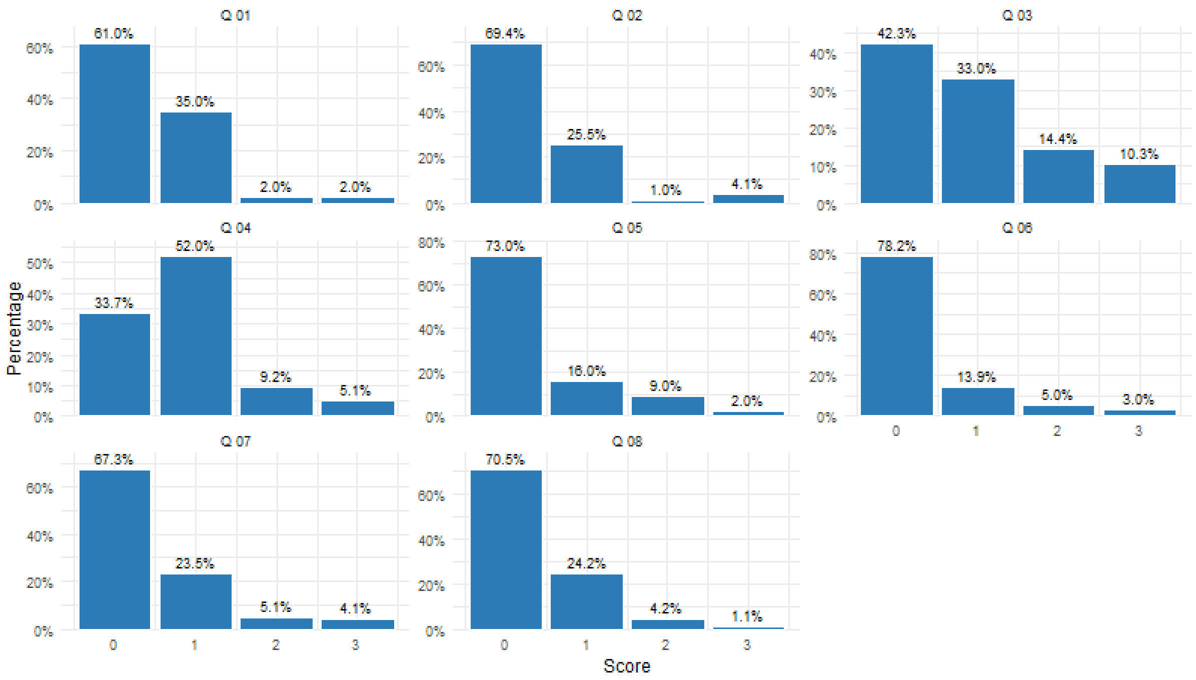

Abbreviations: Q, question.

Figure S5. Distribution of responses to the Patient Health Questionnaire-8 (PHQ-8) items among the study participants. Each panel represents one PHQ-8 item (Q1–Q8), and bars show the percentage of participants reporting each score (0 = not at all to 3 = nearly every day). Higher scores indicate greater severity of depressive symptoms.
